# Targeting Epithelial-Mesenchymal Transition for Identification of Inhibitors for Pancreatic Cancer Cell Invasion and Tumor Spheres Formation

**DOI:** 10.1371/journal.pone.0164811

**Published:** 2016-10-20

**Authors:** Kishore Polireddy, Ruochen Dong, Peter R. McDonald, Tao Wang, Brendan Luke, Ping Chen, Melinda Broward, Anuradha Roy, Qi Chen

**Affiliations:** 1 Department of Pharmacology, Toxicology and Therapeutics, University of Kansas Medical Center, 3901 Rainbow Blvd., Kansas City, Kansas, United States of America; 2 High-Throughput Screening Core Facility, Structural Biology Center, University of Kansas, Lawrence, Kansas, United States of America; University of South Alabama Mitchell Cancer Institute, UNITED STATES

## Abstract

**Background:**

Pancreatic cancer has an enrichment of stem-like cancer cells (CSCs) that contribute to chemoresistant tumors prone to metastasis and recurrence. Drug screening assays based on cytotoxicity cannot identify specific CSC inhibitors, because CSCs comprise only a small portion of cancer cell population, and it is difficult to propagate stable CSC populations *in vitro* for high-throughput screening (HTS) assays. Based on the important role of cancer cell epithelial-to-mesenchymal transition (EMT) in promoting CSCs, we hypothesized that inhibition of EMT can be a useful strategy for inhibiting CSCs, and therefore a feasible approach for HTS can be built for identification of CSC inhibitors, based on assays detecting EMT inhibition.

**Methods:**

An immunofluorescent assay was established and optimized for HTS to identify compounds that enhance E-cadherin expression, as a hallmark of inhibition of EMT. Four chemical libraries containing 41,472 compounds were screened in PANC-1 pancreatic cancer cell line. Positive hits were validated for EMT and CSC inhibition *in vitro* using sphere formation assay, western blotting, immune fluorescence, and scratch assay.

**Results:**

Initial hits were refined to 73 compounds with a secondary screening, among which 17 exhibited concentration dependent induction of E-cadherin expression. Six compounds were selected for further study which belonged to 2 different chemical structural clusters. A novel compound 1-(benzylsulfonyl) indoline (BSI, Compound #38) significantly inhibited pancreatic cancer cell migration and invasion. BSI inhibited histone deacetylase, increased histone 4 acetylation preferably, resulting in E-cadherin up-regulation. BSI effectively inhibited tumor spheres formation. Six more analogues of BSI were tested for anti-migration and anti-CSC activities.

**Conclusion:**

This study demonstrated a feasible approach for discovery of agents targeting EMT and CSCs using HTS, and identified a class of novel chemicals that could be developed as anti-EMT and anti-CSC drug leads.

## Introduction

The American Cancer Society estimated 48,960 new cases and 40,560 deaths from pancreatic cancer in the US in 2015 (>111 deaths per day) [1]. With 6% of 5-year survival, pancreatic cancer has the highest fatality rate among cancers [2, 3]. In addition to late detection and fulminant disease course, the high mortality rate is a consequence of disappointing treatment efficacy [4, 5]. The current standard therapy using the nucleoside analogue gemcitabine produces little impact on median overall survival for patients with locally advanced or metastatic disease, who comprise the majority of cases [6–8]. Recent clinical trials achieved statistical significance adding agents (e.g. erlotinib, or nab-paclitaxel) to gemcitabine, or attempting to develop gemcitabine-free combination regimen (e.g. FOLFIRINOX), but these regimen also added significant toxic side effects [9–14].

Recent studies suggested that within a heterogeneous tumor, a small subpopulation of cancer cells have enhanced capacity to form a tumor, are responsible for propagation, relapse, metastasis, and treatment resistance [15–22]. These cells are referred to as tumor-initiating cells (TICs) or circulating tumor cells (CTCs). Because these cells also possess stem cell-like properties such as quiescence, self-renew, asymmetric division, and multidrug resistance, they are also called cancer stem cells (CSCs) [15]. In the past a few years, CSCs have been isolated from almost every type of solid tumor. In pancreatic cancer, CSCs has been identified that may be the root of the tumor’s high metastasis rate, and the extremely poor prognosis and treatment outcomes in patients [22–24]. Therefore, eliminating CSCs has emerged to be an important step for ultimate elimination of the entire cancer cell population.

Because CSCs are generally resistant to current chemo and radiation therapies [18, 25–30], many cancer therapies, while effective in killing the bulk of tumor cells, may eventually fail because they do not eliminate CSCs, which survive to regenerate new tumors. However, developing drugs preferentially kill CSCs has been a challenge. Few approaches have been described to directly screen for agents that are specifically cytotoxic to CSCs. One reported high throughput screening (HTS) approach is by utilizing a genetically modified breast cancer cell line that has low expression of a cell adherent protein E-cadherin and is therefore forced into a mesenchymal status [31]. Other approaches use suspension culture of tumor spheres as indication of CSCs [32]. The challenge is likely due to two reasons: First, although CSCs can be identified and isolated by cell surface marker profiles, and tumor spheres are cultured *in vitro*, it remains difficult to propagate a stable, undifferentiated CSC population in cell culture suitable for high throughput screening for many solid tumors. Moreover, because CSCs comprise only a small portion of cancer cell populations, standard high-throughput cytotoxic assays applied to bulk populations of cancer cells do not identify agents with CSC-specific toxicity.

Among the biological properties of CSCs, one is that it is highly associated with the phenotypic characteristics identified in the induction of cancer cell epithelial-mesenchymal transition (EMT). EMT is an important initial step during the complicated process of cancer cell dissemination and metastasis, characterized by progressive loss of epithelial markers, such as E-cadherin [33–40]. Induction of EMT in normal or neoplastic cell populations resulted in the enhancement of cells with stem-like properties [41]. Normal and cancer cell populations experimentally induced into EMT exhibited increased resistance to chemotherapies [34, 42]. Recently, a research group has transdifferentiated an epithelial breast cancer cell line into a mesenchymal phenotype by inhibiting E-cadherin expression using short hairpin RNA targeting CDH1 gene (the gene encoding E-cadherin), or forced expression of Twist (transcriptional repressor of E-cadherin) [34]. The breast cancer cells that were induced to EMT exhibited a ~100-fold increase in CSC numbers, and exhibited increased drug resistance to commonly used chemotherapeutic drugs [34]. Remarkably, compounds that selectively inhibited these mesenchymally trans-differentiated cells were subsequently found to inhibit breast cancer stem cells [34]. These studies strongly suggest that it is possible to find compounds with selective toxicity to CSCs by targeting EMT.

Loss of cell surface E-cadherin is a classical hallmark of EMT. E-cadherin primarily expresses in epithelial cells and helps to form a tight, polarized cell layer. E-cadherin also plays an important role in tissue morphogenesis, cell signaling, and cell movement [43]. Studies have shown that loss of E-cadherin was the rate limiting step in the progression from adenoma to carcinoma and the subsequent formation of metastasis [44, 45]. Therefore, we selected E-cadherin as the “readout” indicator for change of EMT in this study. In this study, we aimed to establish a high throughput screening (HTS) assay to simply identify compounds that can restore E-cadherin, in hope to discover inhibitors for pancreatic CSCs. Detecting increase in E-cadherin using fluorescent assay has advantage over detecting loss of fluorescence (e.g. using mesenchymal markers), because decrease/loss of signal can be simply caused by cell death and is not necessarily related to EMT inhibition, and therefore can increase chance of false positive hits. Stem cell markers were not used because their expression are limited in the very small proportion of cells (e.g. CSCs), therefore is not suitable for detection assays used in a high through-put setting. Using a major EMT marker such as E-cadherin is more feasible. We hope the identified EMT inhibitor will hold the possibility of inhibiting CSCs. Even if the EMT inhibitor does not inhibit CSCs, it may hold promises in inhibiting metastasis.

## Materials and Methods

### Cell culture

Human pancreatic cancer cell lines PANC-1, and BxPC-3 were obtained from the American Type Culture Collection (ATCC, Manassas, VA). Human pancreatic cancer cell line L3.6pl was donated by Dr. Liang Xu at the University of Kansas (Lawrence, KS). All cells were cultured in recommended media supplemented with 10% FBS, 100 units/ml penicillin/streptomycin, at 37°C in a humidified 5% CO_2_ atmosphere.

### Immunofluorescent assay for detection of E-cadherin expression

PANC-1 cells were seeded at 10,000 cells/100 μL/well into 96-well plate. E-cadherin was detected by anti-E-cadherin primary antibody followed by Alexa488 conjugated secondary antibody. Fluorescent intensity at 490 excitation/525 nm emission was detected by plate reader. Then the assay was transformed into 384-well plate at the HTS Core at KU Lawrence, seeding 3000 cells/40 μL/well, with 0.35% DMSO as negative treatment control, and 3.5 mM sodium butyrate as positive control. The immunofluorescent assay was automated: Cell seeding, compound treatment, immunostaining, and image capture and analysis were performed with the WellMate® (Thermo Scientific Matrix), the ECHO 550® acoustic liquid handler (Labcyte), the ELx405® CWS Plate Washer (BioTek), the BD Pathway™ 855 High Content Imaging System (Becton, Dickinson), the Nikon Ti-inverted digital microscope (Nikon), and CellProfiler image-based data analysis software (Broad Institute). Automated immunofluorescence staining started following 48 hours treatment by the compounds, and all wash steps were three gentle cycles of PBS addition by WellMate® and aspiration by ELx405®. Cells were fixed with 3.7% formaldehyde and Hoechst for ten minutes, washed, then blocked, permeabilized, and nuclei labelled with 1% BSA, 0.3% TritonX-100. Cells were then treated with anti-E-cadherin primary antibody (1:250 dilution), washed 3 times, followed by Alexa488 conjugated secondary antibody (1:500 dilution) and washing with PBS for 3 times. Nuclei were stained with 2 μg/mL Hoechst 33342. The BD pathway 855 automated microscope (BD Biosciences, San Jose, CA) was used to capture images of cells from four fields per well. Images for each field were acquired in two independent fluorescence channels for Hoechst blue (nuclei) and Alxa488 (E-cadherin). Fluorescence was measured on a cell-by-cell basis, and quantified by CellProfiler software. Subtraction of background staining was performed after image collection using the CellProfiler image-based data analysis software. Some plates appeared to have greater background staining than other plates due to the BD Pathway™ 855 automated gain and exposure settings. The background was taken into account using the post steps in the image analysis in CellProfiler.

### RNA isolation, cDNA synthesis, and Real-Time PCR

Total RNA was extracted from cells by using TRIZOL reagent according to the protocol of the manufacturer (Life Technologies, Grand Island, NY). cNDA synthesis was performed with 1μg of total RNA using Omniscript RT kit according to manufacturer’s protocol (Quiagen, Valencia, CA). cDNA was diluted 1:5 in autoclaved nanopure water. Real-time PCR was performed using BioRad iQ iCycler detection system with iQ SYBR green supermix (BioRad Laboratories, Ltd, Hercules, CA). Reactions were performed in a total volume of 10 μl including 5 μl of 2X iQ SYBR green supermix, 1μl of each gene specific primers at 20 pmol/μl and 1 μl of cDNA template. All reactions were carried out in at least triplicates for every sample. Data were normalized to 18S rRNA.

### Analysis of CD44+/CD24+/EpCAM+ population in PANC-1 cells by FACS

Cells cultured in monolayer were scrapped, washed with PBS, and then block with staining buffer (DPBS supplemented with 0.2% (w/v) bovine serum albumin (BSA) protein, pH 7.4) for 10 min. Cells were triple-stained with phycoerythrin (PE)-conjugated anti-CD24, PE-Cy7 conjugated anti-CD44 and APC-conjugated anti-EpCAM antibodies for 20 min on ice and then were washed and centrifuged with staining buffer thrice. Cells stained with individual antibodies were used as compensation controls. Live cells were analyzed immediately with flow cytometry by using Hoechst 33342 staining for nuclear. PE-Cy7+/APC+ subpopulation in the PE+ gated cells was identified as CD44^+^/CD24^+^/EpCAM^+^ subpopulation.

### Matrigel invasion assay

Cells were seeded into inserts of Boyden chambers (BD Biosciences, San Jose, CA) that were pre-coated or not coated with matrigel (1mg/ml), at 5 x 10^4^ cells per insert in 0.5% FBS containing medium, and then cells were transferred to wells with culture medium containing 10% fetal bovine serum as nutritional attractor. After 24 hrs incubation, invading cells at the bottom side of the insert membrane were fixed with 4% Para formaldehyde for 2 min, permeabilized with 100% methanol for 20 min, followed by staining with 0.05% crystal violet for 15 min at 37°C. Non-invading cells at the top side of the membrane were removed by cotton swab. Photographs were taken from five random fields per insert. Cells in the five random fields were counted.

### Western blot

SDS-PAGE and Western blot was performed as routine. Cells were lysed in ice cold RIPA buffer containing protease inhibitors, followed by sonication and centrifugation. Supernatant was taken for protein quantification by BCA method, and 10 μg proteins were boiled for 10 min before loaded onto 8% polyacrylamide gel for electrophoresis. After the transfer, blockage, and washes, the PVDF membrane was incubated with anti-E-cadherin antibody (1:1000 dilution), at 4°C, for 12 hrs, and then with a goat anti- mouse or rabbit polyclonal horseradish peroxidase (HRP) conjugated secondary antibody (1:10,000 dilution in 1% milk/TBS) for 2 hrs at room temperature. The blots were established by using a chemi luminescence detection kit (Amersham Biosciences, Piscataway, NJ).

### MTT assay

Cells were plated into 96 well plates at a starting density of 10^4^ cells/100 μL/well, and then were treated with drugs. Cell viability was determined after 48hrs of treatment using the 3-[4,5-dimethylthiazol-2-yl]-2,5-diphenyl-tetrazolium bromide (MTT) assay. Absorbance at 570 nm was measured with a kinetic micro-plate reader (BioTek, Winooski, Vermont) and was used as a measure of cell viability.

### Scratch assay

Cells in confluent monolayer culture in 6-well plate were wounded manually by scraping the monolayer using p1250 sterile pipette tip. Debris was washed out with media, and then cells were subjected to treatments. Wound closure was captured using an inverted microscope at 0, 6, 12, 24, 30, and 48 hrs under high power field (100X magnification). The extent of wound closure was calculated as follows: % Distance covered = 100%—(Distance of remaining wound gap ÷ Distance of original wound gap) × 100%.

### Tumor sphere formation assay

Cells in single cell suspension were plated into ultralow attachment 6-well plates (Corning Inc., Corning, NY) at a density of 4,000 cells/well in stem cell media: DMEM supplemented with 1X B27 Supplement, 20 ng/ml human basic fibroblast growth factor, 20 ng/ml epidermal growth factor (Invitrogen, Grand Island, NY), 4μg/ml heparin calcium salt (Fisher, Pittsburg, PA) and 100 units/ml penicillin/streptomycin. Cells were subjected to treatments, and spheres were counted after 14 days using microscopy (primary spheres), and sizes of spheres (Feret diameter) were measured by Image J software. Primary spheres were collected and were dissociated by trypsinization and then passed through a 22G pippetting needle with 90° blunt ends (Fisher Scientific) to obtain a single cell suspension. These cells were seeded and treated the same way again to obtain secondary spheres.

### Statistical analysis

Comparison of data between two groups was performed using t-test. When more than two groups were involved, one way ANOVA with post-hoc test was performed. A difference was considered significant at the p < 0.05 level.

## Results

### Development of HTS assay based on immunofluorescence detection of E-cadherin expression

**Fig 1:**
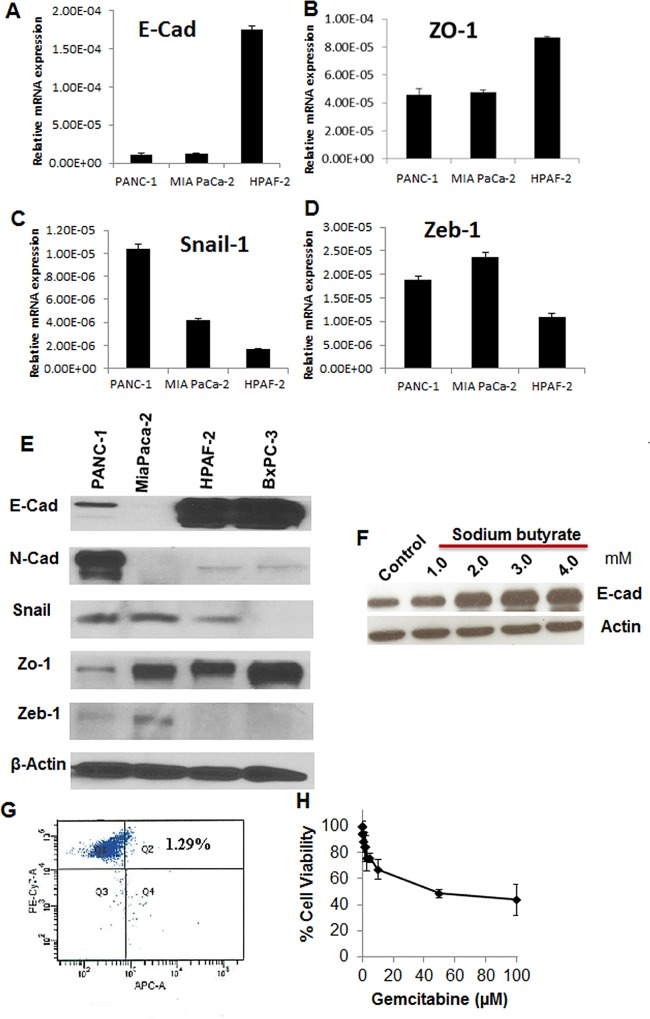
Baseline features of PANC-1, MIA PaCa2, and HPAF-2 pancreatic cancer cells in EMT related gene expression, CSC population, and resistance to gemcitabine. **A-D**. mRNA levels of E-cadherin (E-Cad), Zo-1, Zeb-1 and Snail-1. RT-PCR data was normalized to 18s rRNA and represented as mean ± SD of 2^-ΔCt^ of triplicate determinations of 3 individual experiments. **E**. Western blots for E-cadherin and N-cadherin expression in 4 pancreatic cancer cell lines. Actin was a loading control. **F**. Western blots for E-cadherin expression in PANC-1 cells treated with sodium butyrate. **G**. Flow cytometry identification of CSCs in PANC-1 cells. CD24+/CD44+/EpCAM+ subpopulation were detected as pancreatic cancer CSCs. PANC-1 cells were triple stained with PE-conjugated anti-CD24, PE-Cy7-conjugated anti-CD44 and APC-conjugated anti-EpCAM. DAPI staining was used for identification of living cells. Cells were analyzed with multi-label flow cytometry. The upper-right quadrant showed CD44+/ EpCAM + cells within CD24+ gated population. **H**. Resistance of PANC-1 cells to gemcitabine treatment. PANC-1 cells viability was determined at 72 hrs of incubation using MTT assays. Data represent Mean ± SD of triplicate measurements of 4 individual experiments. Gemcitabine concentrations up to 5 mM were used but failed to achieve 90% cell death.

**Fig 2:**
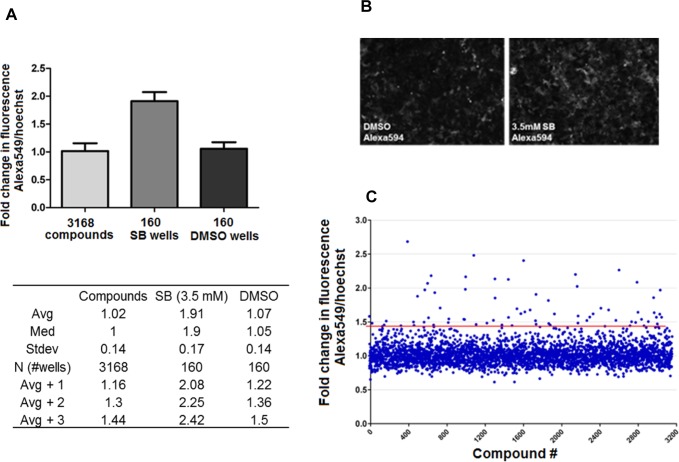
Validation of the immunofluorescent HTS assay. **A**. Average signal and standard deviation of the positive and negative controls, compared to the average of all screened compounds. Total 3,168 compounds were from Microsource (2,320 compounds) and Prestwick (848 compounds) compound libraries. Every plate included 16 wells of vehicle (0.35% DMSO) treated cells, and 16 wells of cells treated with 3.5 mM sodium butyrate (SB). Avg +1, +2 and +3 represent signals 1, 2 or 3 standard deviations above average. **B**. Representative images of E-cadherin immunofluorescence of PANC-1 cells treated by 0.35% DMSO, or 3.5 mM sodium butyrate (SB). E-cadherin was detected by anti-E-cad primary antibody (1:250 dilution) followed by Alexa594 conjugated secondary antibody (1:500 dilution). **C**. The relative fluorescence (ratio of AlexaFluor to Hoechst, fold over DMSO vehicle) was plotted against individual wells to visualize the data spread. The median for control wells (DMSO vehicle) was 1-fold, with a 0.1-fold standard deviation. The HCS assay cutoff was 3 standard deviations above the median (1.437-fold), marked by the red line. Eighty four compounds had readings greater than or equal to this cutoff.

To establish the HTS assay, we selected a pancreatic cancer cell line that possess a mesenchymal-like profile, and has a low expression level but inducible E-cadherin. Four pancreatic cancer cell lines were examined for their basal expression of epithelial (E-cadherin, ZO-1) and mesenchymal markers (N-cadherin), as well as transcriptional repressors of E-cadherin (Snail-1 and Zeb-1) (**Fig 1A–1E**). MIA PaCa-2 had undetectable level of both E-cadherin and N-cadherin but has ZO-1 expression. HPAF-2 and BxPC-3 were highly epithelial in nature with high level of expression of E-cadherin and very low levels of N-cadherin. PANC-1 cells were mesenchymal in nature, as showing by low levels of E-cadherin and ZO-1, and relatively high levels of Snail, Zeb, and N-cadherin, making it potentially suitable for development of the HTS assay. We then examined whether E-cadherin expression could be induced in PANC-1 cells. We used sodium butyrate, a known histone deacetylases inhibitor (HDACi) for this purpose, because sodium butyrate is known to induce E-cadherin expression through HDAC inhibition [46]. Indeed, 1–4 mM of sodium butyrate induced E-cadherin in PANC-1 cells concentration dependently (**Fig 1F**). CSC populations at basal level were detected in PANC-1 cells using multi-fluorescence labeled flow cytometry to identify CD24+CD44+EpCAM+ cells, as these cellular surface markers were indicative for pancreatic cancer stem cells [23]. The triple positive cells comprise ~1.3% of PANC-1 bulk population (**Fig 1G**). Moreover, PANC-1 cells were resistant to gemcitabine treatment, with 100 μM gemcitabine only achieved 60% inhibition (**Fig 1H**). Therefore PANC-1 was selected for screening in high content imager for immunodetection of E-cadherin induction. Sodium butyrate was used as a positive control compound in the establishment of the HTS assay. Immunofluorescent assay was miniaturized for screening in 384-well microplate and was optimized to reduce plate to plate variability, edge effects, and dilutions of primary anti-E-cadherin antibody and a red fluorophore Alexa Fluor 594 conjugated secondary antibody. To reduce possible false positives with the whole plate fluorescence intensity detection, we used the BD Pathway automated microscopy to carry out High Content Screening (HCS), which also took into account nuclei size and shape for simultaneous cell viability. The assay was able to reproducibly detect increases in E-cadherin in PANC-1 cells in response to 3.5 mM sodium butyrate (**Fig 2A and 2B**). Using the HCS, a validation screen was performed on 3,120 compounds drawn from Microsource (2,000 compounds) and Prestwick (1,120 compounds) compound libraries (10 μM). Compounds in these libraries have known bioactivities in several therapeutic areas (marketed drugs and bioactive alkaloids) and represent significant structural diversity. The screen detected 84 compounds with low cytotoxicity (<25% cell loss), which increased fluorescence by 3 standard deviations above average. This represented 2.65% hit rate (**Fig 2C**).

### High throughput screening for compounds that induces E-cadherin expression

**Fig 3:**
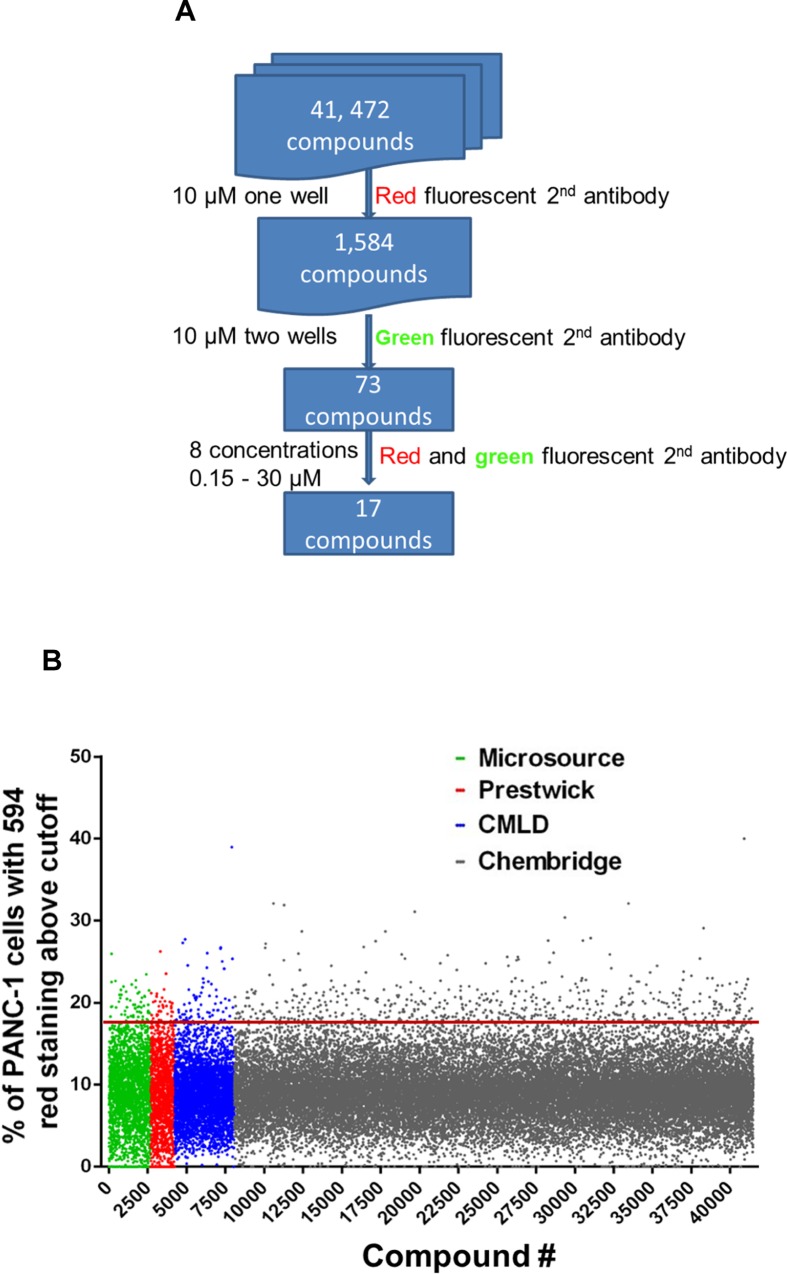
**A**. Scheme of the HTS. **B**. The percent of red fluorescence, relative to DMSO vehicle, was plotted against individual wells to visualize the data spread. The red line represents 3 standard deviations above average.

**Fig 4:**
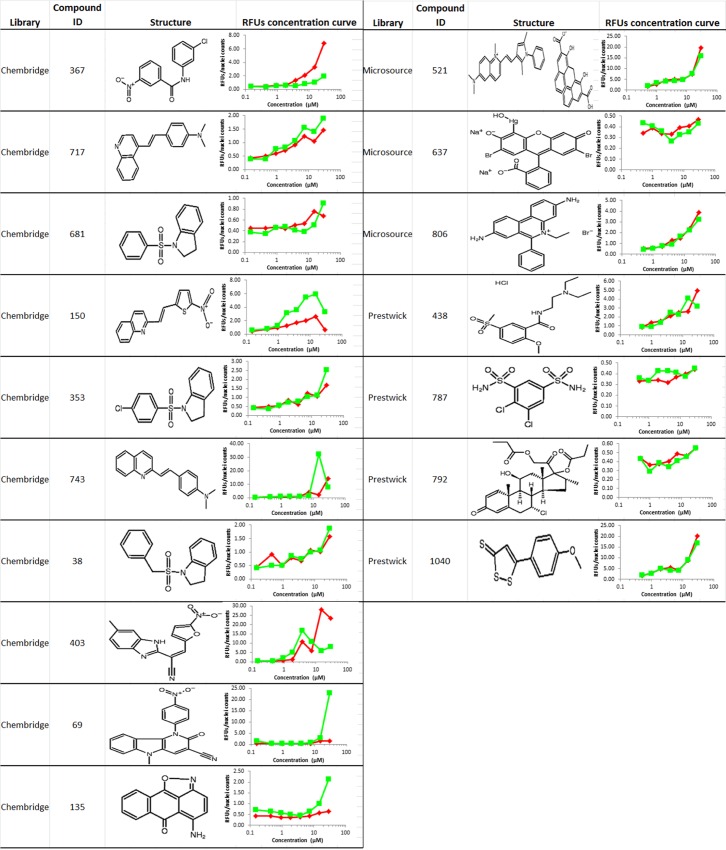
The 17 positive hits. Red curves represent the RFUs using the red fluorophore Alexa Fluor 594 conjugated 2^nd^ antibody in the initial screening, green curves represent RFUs using the green fluorophore Alexa Fluor 488 conjugated 2^nd^ antibody in the secondary screening.

A larger compound library Chembridge library was screened using the optimized HTS assay, which contains 41,472 compounds with structural diversity and drug like properties selected using Lipinski’s Rule of five and other filters (**Fig 3A and 3B**). HTS resulted in 1,500 positive hits at the concentration of 10 μM. To rule out auto-fluorescent compounds that have an emission overlapping with Alexa Fluor 594, we performed a secondary screening using an anti-E-cadherin antibody conjugated to a green fluorophore Alexa Fluor 488. The secondary screening was carried out over the 1,500 initial hits from Chembridge library and the previous 84 hits in the assay validation screening. The secondary screening identified 73 compounds that had fluorescence increase with both red and green fluophore conjugated antibodies. These compounds were then retested at 8 concentrations ranging from 0.15 to 30 μM. Of the 73 compounds, 17 showed dose responsive increases in both Alex594 and Alex488 fluorescence in PANC-1 cells (**Fig 4**). The 17 compounds were checked and passed the pan-assay interference compounds (PAINS) filter using PAINS-Remover (http://cbligand.org/PAINS) for false positive removal [47, 48]. Medicinal chemistry consultation had helped remove compounds that were (1) toxic in other cell based assays, (2) formed crystals in the well, (3) were undesirable chemotypes. Accordingly, there were 6 compounds that passed all of the above filters and were kept in the highest priority list.

### Validation of active compounds for E-Cadherin restoration

**Fig 5:**
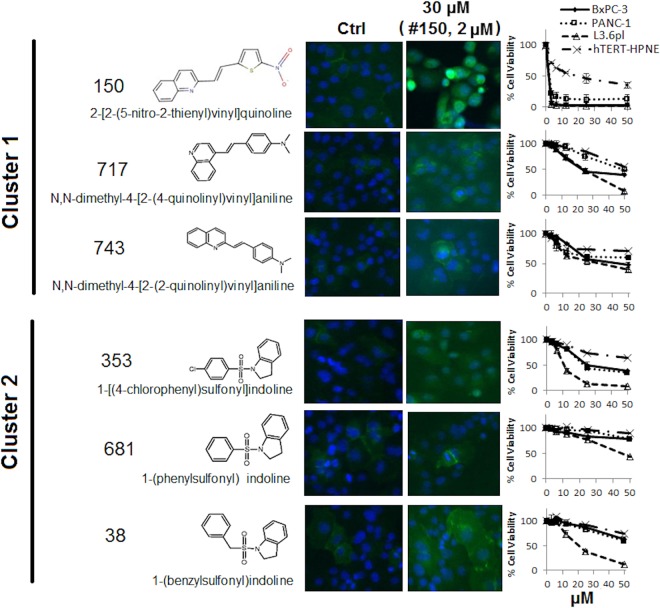
Structure, cytotoxicity and induction of E-cadherin fluorescence by hit compounds. E-cadherin immunofluorescence of PANC-1 cells treated by 6 selected compounds was detected at 24 hrs of treatment by anti-E-cad primary antibody (1:250 dilution) and Alexa 488 conjugated secondary antibody (1:500 dilution). Sensitivity of PANC-1, BxPC-3, L3.6 and hTERT-HPNE cells to the compounds were detected at 48 hrs treatment by MTT assay. Data represents Mean ± SD of 1–3 independent experiments each done in triplicate.

The 6 selected compounds were subjected to further testing, which belong to 2 different structural clusters. Compound 150, 717, 743 are vinyl quinolone compounds (Cluster 1), and compound 038, 353 681 are sulfonyl indoline compounds (Cluster 2) (**Fig 5**). Compounds in both clusters increased fluorescence intensity as detected in the immunofluorescent assay using anti-E-Cadherin antibodies conjugated to Alexa Fluor 488. Compounds in both clusters showed low cytotoxicity (IC_50_ > 50 μM) towards PANC-1 cells except for compound 150 (IC_50_ = 2 μM, in Cluster 1) and compound 353 (IC_50_ = 22 μM, in Cluster 2). Overall, compounds in cluster 1 were more cytotoxic compared with compounds in Cluster 2. Cytotoxicity of the compounds in both clusters was also tested on BxPC-3 and L3.6pl pancreatic cancer cells, and similar results were obtained (**Fig 5**). These hits exhibited comparable or less cytotoxicity to a non-cancerous pancreatic ductal epithelial cell line hTERT-HPNE (**Fig 5**).

**Fig 6:**
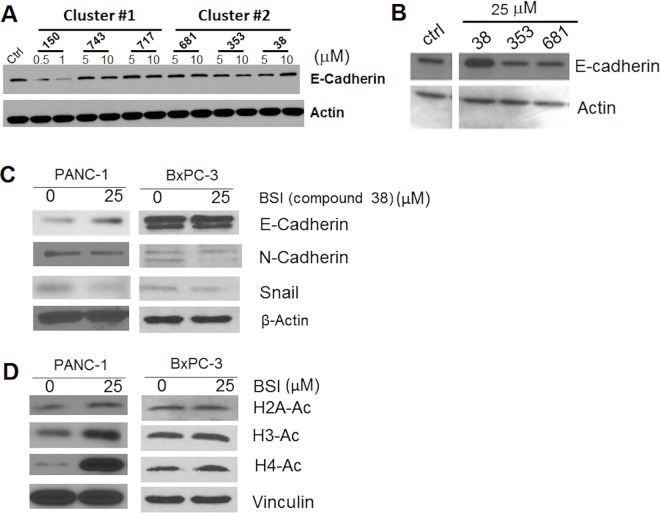
Induction of E-cadherin expression and inhibition of HDACs by hit compounds in PANC-1 cells. **A**. Western blot analysis of E-cadherin in PANC-1 cells that were exposed to two different concentrations of cluster#1 and 2 compounds. Concentrations for compound 150 were 0.5 and 1 μM. **B**. Western blot analysis of E-cadherin in PANC-1 cells that were exposed to 25 μM of cluster#2 compounds for 24 hrs. **C**. Western blot analysis in PANC-1 and BxPC-3 cells for E-cadherin, N-Cadherin, and Snail. Cells were treated with BSI (25 μM) for 24hrs. **D**. Western blot analysis in PANC-1 and BxPC-3 cells for H2A-Lys5, H3-Lys9, and H4-Lys8. Cells that were exposed to BSI (25 μM) for 24hrs.

Ability of these compounds in inducing E-cadherin was further examined by non-fluorescence based western blots. Western blotting in total cell lysate showed that at 5 and 10 μM, compound 38 (1-(benzylsulfonyl) indoline, BSI) in cluster 2 marginally increased E-cadherin expression in PANC-1 cells, and showing a concentration dependence. Compound 150 in cluster 1 decreased E-cadherin protein level at 0.5 and 1 μM probably because of cytotoxicity, and other compounds did not show effects (**Fig 6A**). Increased concentrations in cluster 2 compounds confirmed that compound 38 (BSI) induced E-cadherin expression in PANC-1 cells at 25 μM, a sub-cytotoxic concentration (**Fig 6B**).

### BSI increased E-cadherin expression by inhibition of Snail and HDACs

While E-cadherin was increased, N-cadherin was decreased, and Snail was also decreased in PANC-1 cells treated with 25 μM of BSI for 24 hrs (**Fig 6C**). Consistent results in N-cadherin and Snail decrease were shown in the more epithelial-like pancreatic cancer cell BxPC-3 (**Fig 6C**). E-cadherin was not changed in BxPC-3, probably due to already high baseline level of expression (**Fig 1**). Snail is one of the major E-cadherin transcriptional repressors in pancreatic cancer cells. Snail recruits histone deacetylases (HDACs) to induce epigenetic changes resulting in a closed-chromatin configuration of DNA, and thus inhibits E-cadherin expression [49, 50]. We investigated the HDAC activities by detecting acetylation of histones in PANC-1 and BxPC-3 cells. At 24 hrs of BSI treatment (25 μM), there was a robust increase in acetylation of H4 (H4-K8) compared to untreated cells in both cell lines (**Fig 6D**). There was an increase in H3 lysine 9 (H3-K9) and H2A acetylation in PANC-1 cells but much subtle as compared to H4 acetylation, while in BxPC-3 cells, acetylation of H3 and H2A was not increased (**Fig 6D**). This indicated that BSI has activity in inhibiting HDACs, and preferably induced increase of acetylation in H4. This mechanism is consistent with published data showing that H3/H4 deacetylation silenced E-cadherin [50, 51]. Taken together, BSI (compound 38) was validated as E-cadherin enhancer by inhibiting Snail and HDACs. The selectivity of BSI on HDACs needs to be further studied.

### BSI inhibited pancreatic cancer cell invasion and migration

**Fig 7:**
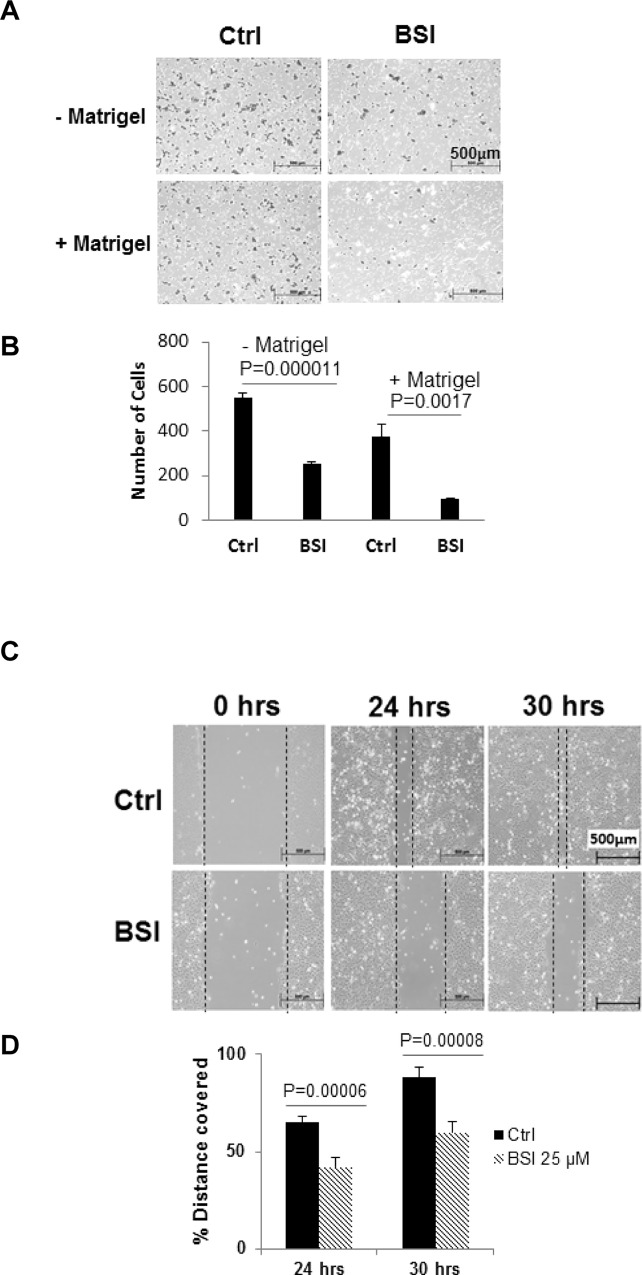
Inhibition in cell invasion and migration of pancreatic cancer cells by BSI. **A, B**. Matrigel invasion assays for PANC-1 cell migration and invasion. Cells were exposed to 25 μM BSI. Cell migration (without Matrigel) and invasion (with Matrigel) were detected at 24hrs. Bar graph (**B**) shows the average number of migrated/invaded cells per field (Mean ± SD of at least 5 fields per experiment for 3 repeated experiments). **C, D**. Scratch assay for BxPC-3 pancreatic cancer cell migration. Scratch was made on confluent monolayer and then exposed to 25 μM BSI. Cell migration was measured at 24 and 30 hrs post BSI treatment. Bar graph (**D**) shows the % distance covered by BxPC-3 cells. Data represents Mean ± SD of 3 repeats.

Boydon chambers covered with Matrigel were used to examine the ability of BSI to inhibit PANC-1 cells invasion, while chambers without Matrigel coverage were used to examine cell migrating ability. Data showed that BSI significantly decreased both invasion and migration of PANC-1 cells at 24 hrs treatment at a sub-cytotoxic concentration of 25 μM (**Fig 7A and 7B**).

Another pancreatic cancer cell line BxPC-3 and a wound healing scratching assay were used to confirm these inhibitory effects. BSI at 25 μM significantly inhibited the ability of BxPC-3 cells to cover the scratched area (**Fig 7C and 7D**).

### BSI inhibited pancreatic tumor spheres formation

**Fig 8:**
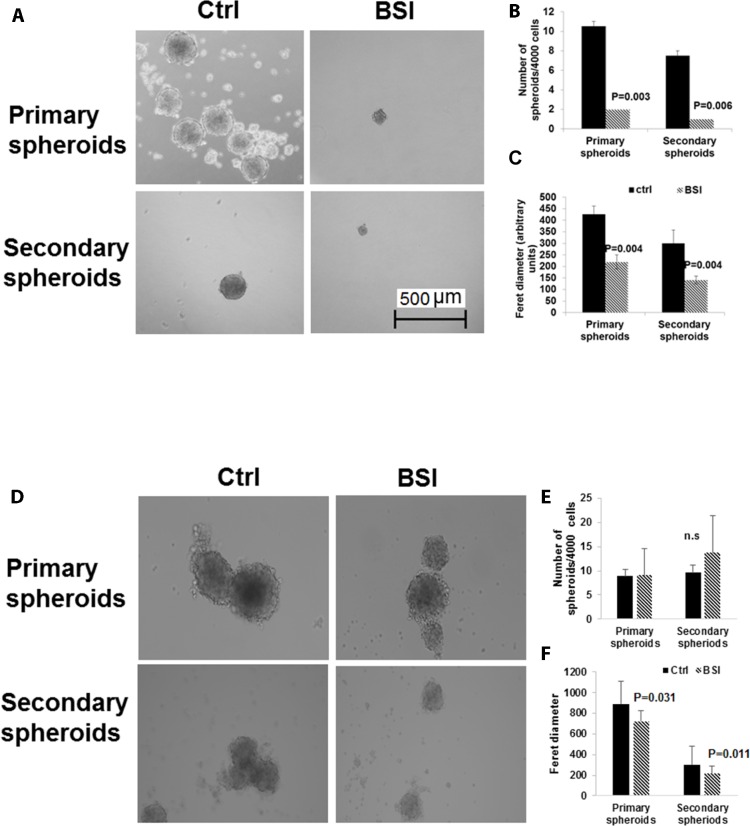
Reduction of the pancreato sphere formation by BSI treatment. BxPC-3 cells (**A, B, C**) and PANC-1 cells (**D, E, F**) were seeded into ultra-low attachment 24-well plates at 4,000 cells/well, and were exposed to 25 μM of BSI. Primary Spheres were imaged and counted 14 days post treatment. Primary spheres were dissociated into individual cells and then reseeded into ultra-low attachment plates for secondary spheres formation. Again cells were exposed to 25 μM of BSI. Secondary Spheres were imaged and counted 14 days post treatment. Scale bar 500 μm. Magnification of the images 100X. Bar graph show Mean ± SD of 6–12 repeats.

The capacity of self-renewal and giving rise to new tumors are the major characteristics of cancer stem cells. In suspension culture, cancer stem cells overcome anoikis (death caused by loss of adhesion) and form tumor spheres, while majority of cancer cells die. Therefore, the number of 3-D tumor spheres in ultra-low adherent tissue culture plates is indicative of the numbers of tumor cells with stem cell-like characteristics [41]. The tumor sphere formation was examined in PANC-1 and BxPC-3 with BSI treatment. In BxPC-3 cells, BSI at 25 μM significantly reduced the number of primary spheres, which indicated reduction in the number of cancer stem cells (**Fig 8A and 8B**). The BSI at 25 μM also significantly reduced the size of each remaining spheres, indicating inhibition also in cell proliferation in the spheres (**Fig 8A and 8C**). The cells in the primary spheres were dissociated into single-cells suspension and treated again with BSI 25 μM under the same condition to account for secondary spheres formation. The number and the size of the secondary spheres were reduced again by BSI treatment (**Fig 8A–8C**). In PANC-1 cells, the number of spheres were not changed by BSI treatment, but the size of both primary and secondary spheres were significantly reduced by BSI treatment (**Fig 8D–8F**).

### Analogues of BSI inhibited pancreatic cancer cell migration, invasion, and tumor spheres formation

**Fig 9:**
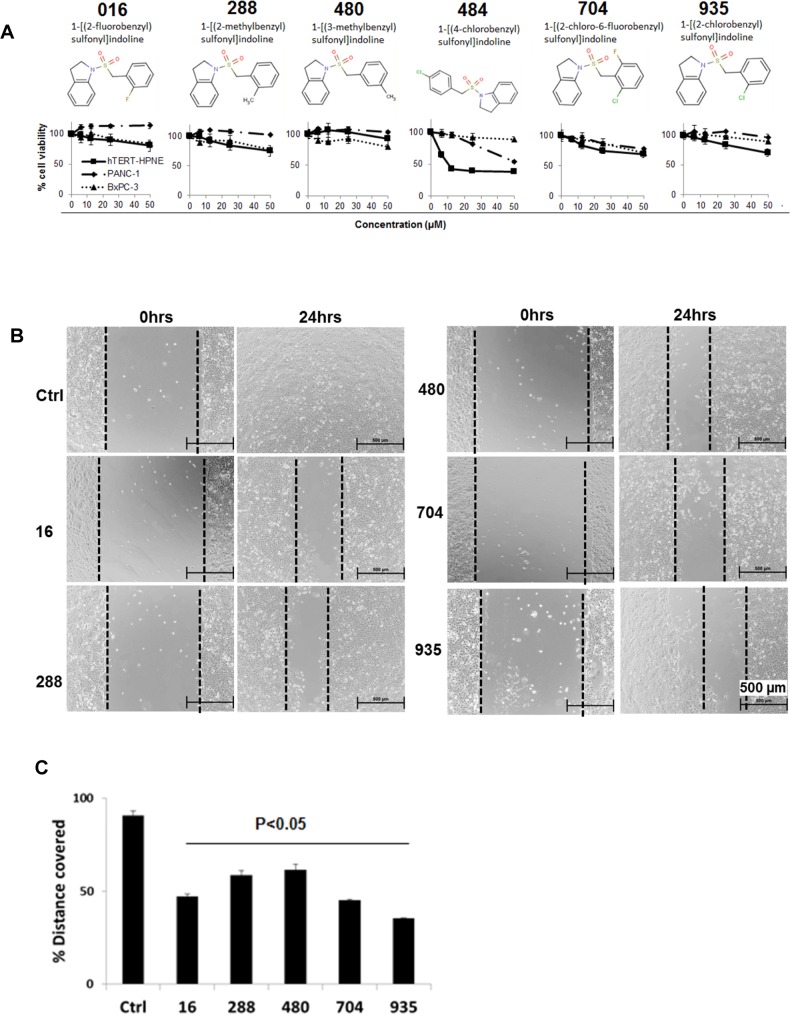
Structure, cytotoxicity and effects of BSI analogues on cell migration. **A**. Structure of the 6 BSI analogues, and sensitivity of pancreatic cancer cells (PANC-1 and BxPC-3) cells to BSI analogues. Cells were exposed to different concentrations of BSI analogues for 48 hrs. Cell viability detected at 48hrs post treatment by MTT assay. **B**. Scratch was made on confluent monolayer of BxPC-3 cells using 1.25 ml sterile pipette tip. After washing with media, cells were exposed to 25 μM BSI analogues. Scratch was photographed at 0 and 24 hrs post treatment. **C**. Bar graph shows the % distance covered by BxPC-3 cells of 3 repeats.

**Fig 10:**
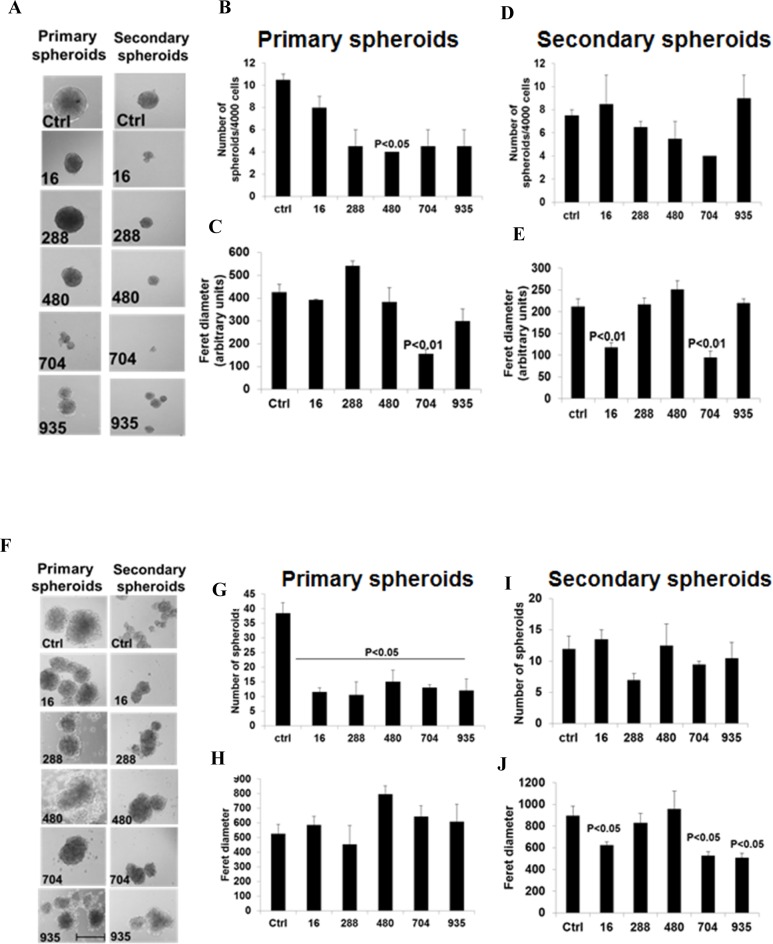
Inhibition of pancreato-spheres formation by BSI analogues. BxPC-3 and PANC-1 cells were seeded in ultra-low attachment plates and then cells were exposed to 25 μM of BSI analogues. BxPC-3 (**A**) and PANC-1 (**F**) primary spheres were imaged and counted 14 days post treatment. Bar graph representing the average number of primary spheres of BxPC-3 (**B**) and PANC-1 (**G**), feret diameter of the primary spheres of BxPC-3 (**C**) and PANC-1 (**H**) spheres± SEM. Primary spheres were dissociated into individual cells using trypsin and then reseeded into ultra-low attachment plates for secondary spheres. Again cells were exposed to 25 μM of BSI analogues. BxPC-3 (**A**) and PANC-1 (**F**) Secondary Spheres were imaged and counted 14 days post treatment. Bar graphs representing the average number of secondary spheres ± SD of BxPC-3 (**D**) and PANC-1 (**I**) cells, feret diameter of BxPC-3 (**E**) and PANC-1 (**J**) spheres. Scale bar 500 μm. Magnification of the images 100X.

Six more analogues of BSI were purchased from Chem Bridge Chemical Store (**Fig 9A**) and tested for their effects on pancreatic cancer cell migration, invasion and tumor spheres formation. All of these compounds possessed low cytotoxicity towards PANC-1 and BxPC-3 cells, as well as to the non-cancerous pancreatic ductal epithelial cell hTERT-HPNE (**Fig 9A**). Compound 484 was more toxic to the normal cell hTERT-HPNE than to the cancer cells, and therefore was excluded for further tests. Compounds 16, 288, 480, 704 and 935 inhibited BxPC-3 cell migration at 25 μM, as evaluated by wound healing scratching assay (**Fig 9B and 9C**). Their activities were comparable in inhibiting cell migration (**Fig 9C**), At 25 μM, all of the 5 compounds exhibited inhibitory effect towards primary and/or secondary tumor spheres formation in both BxPC-3 (**Fig 10A–10E**) and PANC-1 cells **(Fig 10F–10J**). However, none of these analogues had superior effect than BSI in reducing numbers or sizes of tumor spheres. These analogues did not show superior inhibitory effects compared to BSI on cell migration/invasion and tumor spheres formation.

## Discussion

Challenge in discovering CSC inhibitors co-exists with challenge in treatment for pancreatic cancer, as CSC is proposed to be the driving force of the tumor’s high metastasis rate and recurrence rate, which directly resulted in extremely poor prognosis and treatment outcomes for patients [22–24]. As direct screening is difficult for CSC-toxic compounds, our study provided an alternative in screening for EMT inhibitors, as an initial step for development of CSC inhibitor. The rationale roots in the close association between EMT and CSC, as studies showed many genes, transcriptional factors and signaling pathways that induce EMT are also important in CSC transformation and maintenance, such as Wnt and Notch pathways [52, 53]. Induction of EMT resulted in increased drug resistance, metastasis, and increased number of CSCs. Therefore, looking for EMT inhibitor holds the hope of also discovering CSC inhibitor. Furthermore, because loss of E-cadherin expression is associated with metastasis in many neoplasms [54–57], EMT inhibitors can stand alone for anti-metastasis, even if they do not inhibit CSCs. As a proof of this concept, our screening for E-cadherin inducers resulted in identification of the novel compound BSI as potent EMT inhibitor. BSI inhibited pancreatic cancer cell migration/invasion, also strongly inhibited CSCs.

Loss of E-cadherin, as a classic hallmark of EMT, can occur by either genetic or epigenetic alterations [58, 59]. Recent reports suggested that histone acetylation regulated E-cadherin expression. Transcriptional repressors of E-cadherin, i.e. Snail1/2 and Zeb1/2, recruit histone deacetylases (HDACs) to conduct an epigenetic alternation that inhibit E-cadherin gene transcription [60, 61]. Our hit compound BSI substantially increased Histon-4 acetylation, indicating HDACs inhibition as its mechanism of action. Moreover, BSI showed selectivity on increasing H4 acetylation compared to the other histones H3 and H2A. This could be an advantage as many of the clinical side effects of HDAC inhibitor is due to lack of selectivity of the inhibitor. Some patent data suggested that compound with structure similar to BSI have serine protease inhibitory effects [62], raising the possibility that the changes in E-cadherin and inhibition in migration/proliferation was due to off target protease inhibitor actions versus specific actions through E-cadherin. Therefore, we examined the expression of P53, a signaling protein that is not associated with E-cadherin pathways. There was no change in P53 expression in both PANC-1 and BxPC-3 cells treated with BSI (data not shown). Except for increase in protein (e.g. E-cadherin), decrease in some proteins were also detected with BSI treatment, such as N-cadherin and Snail, indicating the effects of BSI were not likely due to the protease inhibition. Other mechanisms are not excluded for BSI’s activity in inhibiting CSC. Further in-depth mechanistic studies are worth carrying out, given the anti-CSC potential of this cluster of compounds.

It is proposed that the functional group in known HDAC inhibitors are either hydroxyamic acid or benzamide groups [63]. BSI and its analogues studied here do not contain these structural groups. Instead, they are benzyl sulfonyl indoles. These data revealed a new class of potent HDAC inhibitors that possesses *in vitro* anti-EMT and anti-CSC activities, with low cytotoxicity. Novel drugs and drug leads can be developed based on further studies on the structure-activity relationship (SAR). Because these compounds were not generally cytotoxic, safety and low toxicity could be expected in future *in vivo* studies.
